# Urogenital fistula reviewed: a marker of severe maternal morbidity and an indicator of the quality of maternal healthcare delivery

**DOI:** 10.1186/s40748-015-0020-7

**Published:** 2015-08-19

**Authors:** Margo S. Harrison, Hillary Mabeya, Robert L. Goldenberg, Elizabeth M. McClure

**Affiliations:** Department of Obstetrics/Gynecology, Columbia University, New York, NY USA; Moi University School of Medicine, Eldoret, Kenya; RTI International, Durham, NC USA

**Keywords:** Urogenital fistula, Obstetric fistula, Maternal morbidity, Low and middle income countries

## Abstract

**Background:**

While obstetric fistula has been recognized as a major maternal morbidity since the 1980s, it has become an indicator of access to and quality of women’ s health care.

**Findings:**

Obstetric fistula still exists in low-income countries (LIC) because health care systems fail to provide adequate family planning, skilled birth attendance, basic and emergency obstetric care, and affordable treatment of fistula, while concurrently lacking social networks to serve as safety nets for affected girls and women [WHO, 2007].

**Conclusion:**

This review explores the most recent published experience with respect to the definition of fistula, its diagnosis, treatment, and management, and further steps for prevention of fistula on a global scale.

## Introduction

An urogenital fistula is defined as an abnormal communication between the bladder, ureter, urethra, vagina, and/or rectum with resulting incontinence of urine and/or stool. It may occur as a sequela of childbirth or as a result of surgical injury, malignancy, infection, trauma, or endometriosis. The term obstetric fistula refers to fistulae resulting from the ‘obstructed labor injury complex’, which describes the injury that occurs when the presenting fetal part becomes impacted against the bony pelvis during labor causing hypoperfusion of the soft tissues in between, resulting in ischemia, necrosis, and an abnormal communication between two pelvic organs [2]. Not all obstetric fistulae result from obstructed labor. A recent review of almost 6,000 cases of urogenital fistula suggested that just over thirteen percent of fistulae were iatrogenic, 80 % of which followed surgery for obstetric complications including cesarean section (57 %), repair of ruptured uterus (20 %), and hysterectomy for ruptured uterus or obstetric indications (3 %), with the remainder occurring during gynecologic surgery unrelated to pregnancy [3]. Of note, urogenital fistulae are often referred to by the organs they involve; for example, a vesicovaginal fistula involves the bladder and vagina, rectovaginal the rectum and vaginal, ureterovaginal the ureter and vagina, and vesicouterine the bladder and uterus. Urogenital fistulas are quiet varied and can include any and/or all of the genitourinary system, but those involved are often reflected by how the fistula is named.

### Epidemiology, incidence, and prevalence

With the recognition of urogenital fistula as a public health indicator related to both the availability and quality of women’s healthcare services, there has been much interest in determining its incidence and prevalence around the world. Fistula patients are a literal embodiment of healthcare system failure to provide appropriate maternal healthcare and delivery services, and as such, fistula occurrence can be used as a measure to determine the quality of the healthcare system in a given area. Commonly quoted numbers suggest that there are 3.5 million women currently living with urogenital fistula and that 50,000 to 100,000 women develop fistula annually [4, 5]. A recent meta-analysis estimated a pooled prevalence of 0.29 fistulae per 1000 reproductive age women in all regions with a rate of 1.6/1000 in sub-Saharan Africa and 1.2/1000 in south Asia [6]. The pooled incidence was 0.09 fistulae per 1000 recently pregnant women [6]. Prior to this publication, the previously accepted numbers regarding incidence came from the 2000 Global Burden of Disease report that suggested the incidence was 0.08 % of all births and 2.15 % of births complicated by neglected obstructed labor [7].

Current methods for assessing the prevalence of urogenital fistula include self-reporting and general communication with surgeons, studies by advocacy groups, and reviews of hospital services; they mostly use community or facility sampling or a combination of both [8]. Because of the relative rarity of fistula, the desire of affected women to hide their condition, and the poor quality of data collection methods in areas where women are affected, obtaining quality data is very difficult [8]. A recent review published on determining the worldwide incidence and prevalence of urogenital fistula suggests that data need to be collected through routine surveillance and monitoring systems that are currently integrated into established health systems and national programs [8]. An example given is the Demographic and Health Surveys that many countries conduct, which include home births; questions should be added to the survey regarding urogenital fistula to garner better data [8]. Additionally, follow-up data collection on women seeking care is also important to track the met need for surgical care and to observe how targeted prevention or treatment interventions are affecting outcomes related to urogenital fistula [8]. The aforementioned review also suggests that fistula care be part of the full continuum of maternal health services provided in maternity wards, and that providers should be trained to assess for urogenital fistula at postpartum visits [8].

The global public health community has recognized the necessity of good data collection regarding urogenital fistula not only in terms of incidence and prevalence, but also in terms of availability of centers for fistula care and providers capable of providing high quality treatment. As such, the WHO developed the Global Fistula Map in an attempt to show not only where women are affected around the world, but also where providers are available [9]. The map was unable to be reproduced for this review, but can be accessed online at http://www.globalfistulamap.org/. Information on how the data was collected is explained on the website and the actual data itself can be downloaded for review.

### Risk factors and causes

Obstetric fistula is associated with age < twenty years, first pregnancy, labor greater than 24 h, delivery at home, height < 150 cm (<59 in.), low levels of maternal education, poor contraceptive utilization, low rates of antenatal care utilization, and having a male fetus [10–12]. A given woman’s risk for obstetric fistula formation is also determined by her overall health, socioeconomic status, and her access to and utilization of health care services [11]. While the immediate cause of obstetric fistula is the lack of safe labor and delivery services, root causes include poor health care infrastructure such as transportation and communication, as well as cultural norms that devalue women, including lack of female autonomy, economic and social independence, and education [11].

### Socioeconomic and psychosocial implications

Urogenital fistula follows acute physical trauma, but it causes continued physical and psychosocial trauma through exclusion from social networks, divorce, impoverishment, and major depression [12]. Role loss, isolation, and economic deprivation were the most common consequences for women with fistula, and the majority of these women were abandoned by husbands, shunned by family, and struggled severely with hygiene associated with incontinence and wound care, as well as frequent infections [13]. The women suffer from post-traumatic stress disorder, social isolation, feeling like the object of stigma, low quality of life, and general mental health dysfunction, including suicidal ideation [14–16].

## Diagnosis, treatment and management

### Timing and symptomatology

Women with urogenital fistula classically present with continuous urinary incontinence, as compared to incontinence with a valsalva maneuver, or stress urinary incontinence, which is common after normal labor and delivery [17, 18]. While timing of presentation after delivery can be affected by socioeconomic as much as clinical factors, it can also be determined by the type of fistula from which a woman suffers. For example, fistula from cesarean delivery may present seven to ten days post-operatively due to formation of a fistulous tract over time, while obstetric fistula due to obstructed labor can usually be noted immediately after delivery [1, 19, 20]. Fistulas that involve the uterus can involve irregular bleeding patterns and loss of blood in urine [19, 21]. Additionally, if the ureter is transected, the presentation will likely occur immediately post-operatively as an intra-abdominal urinoma when the patient begins to experience the pain, pressure, and symptoms produced by the mass effect of extravasated urine, but may not present as a fistula until the anomalous tract has formed with the vagina or another body cavity [19].

### Diagnosis

Diagnosis involves a full medical and social history, including details about the incident pregnancy, the labor course, the delivery, and the fetal outcome. Review of systems should be performed with attention to urinary and fecal symptoms, as well as general mobility and musculoskeletal function [1]. The physical examination begins with collecting vital signs and performing a neurologic and gait evaluation [1]. Visual inspection should always be the first step in the genital exam with evaluation for perineal dermatitis, ulceration, infection, and scarring from prior episiotomy, circumcision, or fistula repair [1]. The examiner should then proceed to abdominal and bimanual examination noting the severity and nature of vaginal and rectal scarring; location, size, and number of fistulae; and involvement of crucial structures such as the urethral sphincter, anal sphincter, and urethra itself [1]. Further diagnostic testing may be necessary, such as a vaginal speculum exam for improved exposure, or a dye test [22]. Dye tests generally involve back-filling the bladder and temporarily occluding the urethra for evaluation of dye leakage. If the fistula cannot be immediately located, some authors recommend vaginal packing, with the location of dye leakage on the pack used to help narrow down the fistula position [17]. Additionally, oral pyridium or intravenous indigo carmine can be administered to assess ureteral involvement [17, 19]. Hemoglobin determination and testing for sexually transmitted infections are also recommended, as the former is important for pre-operative planning, and the latter because sexually transmitted infections can be the cause of a fistula (and their treatment may allow for spontaneous fistula closure), and they can also cause friability of the tissue which will complicate fistula closure. Additionally, patients who are HIV positive may require treatment and rehabilitation prior to surgery to improve their surgical outcomes.

### Treatment

The primary goal of fistula treatment is continence. Obtaining continence generally requires surgery, but may be achieved by conservative treatment with catheters or stents, or require a more aggressive diversionary procedure [1, 23].

### Conservative management

Women with a small simple fistula discovered shortly after delivery, or those presenting to a facility with obstructed labor, may be treated conservatively with insertion of a Foley catheter for somewhere between two and six weeks, twice daily sitz baths, high volume oral intake of fluids, and treatment of any obvious concurrent infections [1]. Currently available data are of poor quality and no clear recommendations can be made as to whether initial conservative management with a Foley catheter is appropriate or effective [24]. However, since 1942, papers have been published suggesting use of a Foley catheter as a method of assisting spontaneous closure of vesicovaginal fistulae less than one centimeter in size [24]. Similarly, ureteral stents placed for one to two months may result in spontaneous resolution of fistulas involving the ureter in greater than half of cases [17, 23]. Ureteral stents are usually placed, in this context, by way of cystoscopy, which may not be available in LIC.

### Surgical management

For most patients, surgery is the only option. The overriding principle of fistula repair is that the first attempt offers the best chance of successful closure [1]. The basic principles of surgical repair are: 1) achieving adequate exposure, 2) mobilizing the fistula from surrounding scar tissue, so that 3) a tension-free closure can be performed that is water-tight [1, 4].

### Timing of surgery

While the traditional teaching is that patients undergo surgery three months after diagnosis to allow time for the fistulae to become less inflamed, more recent data suggest that fistulae be repaired immediately if diagnosed within 72 h of delivery, or even within the three month window, as repair within this timeframe prevents a significant amount of the negative social, economic, and physical sequelae associated with incontinence [17, 19, 23, 25]. Some patients, however, may present years after fistula formation. For these patients, if there is no residual edema, erythema, or persistent granulation tissue, and no need for treatment of infections, anemia, or malnutrition, surgery can be pursued without additional delay [19]. Iatrogenic fistulae should undergo surgical repair with diagnosis, unless the fistula is the result of retained suture. In that case, waiting until the suture is resorbed is in order [3, 19].

### Pre-operative care

The WHO advises pre-operative management include anesthesia evaluation, skin preparation, hair clipping, high oral fluid intake, bowel preparation, and nothing by mouth from midnight of the night before surgery [1]. Issues such as nutritional supplementation and pre-operative estrogen usage, as well as transfusion or supplementation for anemia and empiric treatment with anti-malarials, antibiotics, or antiparasitics are topics for further research.

### Surgical methods

The WHO guidelines recommend a vaginal approach to urogenital fistula repair. The optimal surgical position is high lithotomy, and the optimal anesthetic technique is regional [1]. Vaginal repair is associated with less blood loss, results in shorter operative time, leads to decreased use of analgesics, and overall accounts for shorter hospital length of stays [19]. Various antibiotic regimens, which include single-dose gentamicin, appear to be equally effective at decreasing post-operative urinary tract infections and improving leakage and incontinence profiles on discharge from the hospital [19, 26]. Commonly performed initial surgical steps include episiotomy for exposure, placement of retractors that assist with visualization, Foley catheterization to divert urine from the operative field, protection of the ureters with stents, and placement of a probe into the fistula to delineate its course and establish its boundaries [17].

Closure methods include the Latzko technique (partial colpocleisis without excision of the fistula tract), layered closure (excision of the fistulous tract), and use of flaps, which may be biologic (such as the Martius flap—a labial fat pad), but also include more recent experimentation with synthetic materials [17, 19, 23]. Studies have shown the Latzko technique to be quite effective with success rates quoted as 93 to 100 %, and placement of the Martius flap to have 70 – 100 % success rates, with the caveat that the latter method is used in the setting of more complicated fistulae with greater fibrosis and necrosis, or lack of tissue available for closure [19]. There is also a role for more invasive procedures that necessitate an abdominal approach for patients with reduced bladder capacity or pliability, involvement of the ureter, trigone, ureteral orifice, or cervix, and inability to access the fistula by way of the vagina, or for patients who have not achieved continence after multiple repairs or those whose fistula is too large, or the remaining tissue is too scarce, that anatomic closure is not possible [17, 27].

### Minimally invasive surgery

Although minimally invasive surgery (MIS) such as laparoscopy and robotics is more accessible in high-income countries (HIC), a group in India is utilizing MIS to repair urogenital fistulae resulting from obstetric complications [28]. This group reportedly closed vesicovaginal fistulae with a single layer continuous laparoscopic suture with interposition of an omental flap; urethral catheters were left in situ for a month post-operatively [28]. As the capacity for MIS in LIC builds, it will be interesting to see how this experience contributes to the surgical literature.

### Post-operative care

The WHO guidelines recommend regularly scheduled vital signs, pad checks and catheter monitoring for genitourinary bleeding, intravenous fluids, strict tracking of intake and output of fluids, and regularly scheduled analgesia for pain control, which will allow earlier patient mobility [1]. Patients are encouraged to continue to maintain very high fluid consumption in the early days following surgery, and the catheter is advised to be left in situ for a minimum of 10 – 14 days, with removal of any necessary vaginal packing after 24 – 72 h [1, 19, 26]. There are no recommendations regarding post-operative antibiotic use, but some research suggests that if used, antibiotics should cover all vaginal flora [17].

## Outcomes

No straightforward framework exists for analyzing the determinants of successful fistula treatment and outcomes. This also applies to fistula classification—no generally accepted method exists: there are currently 25 proposed systems in practice, none of which were developed based on empiric evidence or with prognosis in mind [29]. Standardization of terminology regarding urogenital fistula will not only allow development of an evidence-based prognostic classification system, but will also facilitate research, guideline development, and the analysis of clinical outcomes to determine the safety, efficacy, and quality of preventative, diagnostic, and treatment interventions [24, 30]. Even the definitions of ‘success’ and ‘failure’ of fistula treatment are poorly defined; many studies define success as physical closure of the fistula while others define success as continence immediately after surgery and then at a later point in time. These assessments are made by back-filling the bladder with dye and evaluating leakage and continence after catheter removal; the time point at which this exam is performed is usually at surgery to confirm fistula closure, and then two weeks post-operatively.

Despite the lack of uniform definitions of type of fistula and measures of success, if success is defined as physical closure of the fistula, then the literature reports a 55 % to 95 % closure rate with an average success rare of about 85 % [30]. If success is defined as urinary continence then outcomes range from about a 40 % to 90 % success rate with an average success rate of about 70 % [30]. According the WHO guidelines, when establishing a fistula treatment program, it is expected that the closure rate for fistula should be 85 % and the continence rate should be 90 % after a patient’s first fistula repair surgery [1]. Clinical factors that can affect surgical outcome and success rates include degree of urethral involvement (some damage versus circumferential involvement); size, location, and number of fistulae; amount of scar tissue and remaining healthy tissue, including bladder capacity; and whether or not the patient has previously undergone repair [30].

### Psychosocial

Beyond documenting the distress experienced by fistula patients, researchers are implementing interventions to improve the mental health of this population. A Tanzanian trial of a six-session treatment plan based on psychological theory (cognitive behavioral therapy) was integrated into the clinical flow of a fistula ward (two sessions pre-operatively, four sessions post-operatively) and conducted by a non-specialist mental health provider, to improve mental health outcomes for fistula survivors [31]. The ratings from participants were very positive. While that study worked with patients individually, other studies have looked at group therapy and also achieved measureable success [32]. The data suggests that mental health interventions are very important for fistula patients and can be executed concurrently with surgical treatment during the time when patients are preparing for and recovering from surgery [31].

### Physiotherapy

The WHO practice guidelines recommend including physiotherapy as part of the treatment program (deep breathing, hip stretches, lower extremity range of motion stretches, and core strengthening exercises) as well as post-operative workouts that include sitting, standing, walking, and balancing, with graduated strength training, and a detailed program of special movement, positioning, and passive stretches for patients affected by contractures and nerve injuries [1]. A study of pre- and post-operative health education and physiotherapy of urogenital fistula patients reported that those who underwent physiotherapy were almost three times more likely to recover with less post-operative stress incontinence, and in a follow-up study conducted by the same group a year later, the results were maintained and quality of life had significantly improved [33, 34].

### Socioeconomic practices & reintegration

The guiding principles for reintegration programs are that patients should be taught self-sustaining skills, which may require literacy training or workshops in making clothes or crafts. These skills become especially important for women who are single and no longer have the ability to bear children or function as homemaker, and who otherwise have no one to support them. They should be offered counseling services and should be helped to reintegrate into their social support network, which includes their communities and families, but might also involve enrollment in a women’s or fistula survivors support group [1]. Currently utilized interventions include training in income generating skills, literacy training, and microcredit programs, as well as post-operative financial support in the form of clothing, supplies, food, water, and actual stipends [35]. While data in the area of economic repercussions of fistula are lacking, recent research from Tanzania reported that fistula patient’s hopes and concerns about the future are primarily related to their ability to work, in addition to social acceptance and future fertility [13, 36, 37].

### Post-fistula

Some data exist on long-term follow-up of fistula patients after repair. A Nigerian study evaluated 150 women six months after repair to assess quality of life, physical health, mental health, social health, and environment (available income, ability to perform activities of daily living, and level of participation in leisure activities), comparing those indicators before and after surgery; the results were significantly improved on all measures except ‘environment’ [38]. A study of Ethiopian women reported that, the majority felt a dramatic sensation of relief and happiness following repair, yet some continued to experience mental anguish, stigma, and physical problems regardless of the outcome of the procedure. All women suffered intense fear of developing another fistula, most commonly from sex or childbirth. Despite this, the majority of women had sex or planned to, while a smaller cohort avoided intercourse and childbearing, thus subjecting them to isolation, marital conflict, and/or economic vulnerability [39].

A study of fertility in 32 women with fistula from Malawi showed that about half of pregnancies conceived with an active fistula, and 70 % of those conceived post-repair, ended in spontaneous abortion or perinatal death [40]. Findings suggest that pregnancies conceived post-repair may result in poor outcomes years out from surgery; whether these results are related to fistula or occur from coincident damage to the pelvis or some other etiology is not clear. Data on contraception use in women with fistula or post fistula repair is scarce. However, a study of almost 200 women in Nigeria, showed that almost all knew about contraception, but less than half actually utilized it, suggesting that contraceptive uptake after fistula repair was poor for fear of adverse effects (41 % of participants), desire for fertility (30 %), religious prohibition (22 %), cultural beliefs (25 %), and partner disapproval (36 %) [41]. No studies were found on interventions providing contraceptive counseling to urogenital fistula patients.

## Findings

### Prevention & awareness

Whether the fistula results from poor labor and delivery care or poor surgical technique, both are avoidable, and the key is prevention. Improved access to high-quality emergency obstetrical care including cesarean section is essential, but so is improved access to family planning services [25]. Over the longer term, attention should be devoted to developing programs that combat the root causes of fistula formation including improved education, economic opportunity, and gender equality for women [25]. While many of the factors leading to fistula formation may be out of the control of the individual woman, the decision to seek care for a dysfunctional labor falls to the patient and birth attendant. Seeking timely care for prolonged labor is a critical component of obstetrical fistula prevention programs in resource-limited settings [25]. To achieve the goal of overcoming this initial delay to care, patients must value the services provided by health institutions and understand the consequences of not seeking care in a time-sensitive fashion. To accomplish this, the care women anticipate receiving must be effective and of high quality, and be socially, physically, and economically accessible. Overcoming these barriers requires utilization of prenatal care and education of men and the greater community regarding dysfunctional labor and the risk of fistula [25].

A recent review article examined current evidence supporting fistula prevention strategies in sub-Saharan Africa [42]. The authors advise promoting a minimum of post-primary education for girls; providing sexual education that includes information about fistula; educating communities about cultural, social, and physiological factors that influence and contribute to fistula; delaying early marriage and childbirth; eradicating malnutrition; and defining time limits for labor at home without progress [42]. The health system based strategies that have shown success in prevention of fistula include scaling up access to, availability of, and provision of emergency obstetric care; provision of affordable, safe, and timely interventions for women in need of care; reducing the distance to access care; and providing affordable transportation to health facilities [42]. Clearly, additional training or re-training of surgical providers is also crucial, since iatrogenic fistulae due to poor surgical technique are also preventable.

In terms of actual interventions to prevent fistula that have been published, there exist a few. One author applied a fistula index he developed, which is the multiplication of a given patient’s height in centimeters by her intertuberous distance (the distance between the ischial tuberosities of her pelvis), which was measured by the number of knuckles on the surgeon’s hand that he could fit between the pelvic bones [43]. He applied his index in a case-controlled study of 39 fistula patients and 54 controls with normal vaginal deliveries [43]. His results were significantly different between the groups, which was not the case when height alone was compared, suggesting that such a measure of clinical pelvimetry could be useful in identifying women at risk for fistula development [43]. Another study conducted in Niger employed a community-mobilization program to reduce maternal and neonatal mortality and reduce urogenital fistula formation [44]. The intervention utilized village volunteers to identify and evacuate women with protracted labor, provide education, and collect data on pregnancies, births, and deaths [44]. Over three years the intervention significantly reduced maternal and perinatal mortality and reduced the occurrence of fistula from seven cases in the first six months of the study to zero cases in the next 24 months [44]. These interventions suggest that some combination of prenatal risk assessment and education with access to emergency obstetrical care can reduce and potentially prevent urogenital fistula from obstetric causes.

### Training & facility capacity

Even though efforts have been underway to address the problem of urogenital fistula on a global scale for at least 30 years, it was not until 2003 that the United Nations established the Campaign to End Fistula, and it was not until almost ten years later that attention was paid to issues such as the availability of providers and facilities to address the issue. Thus, WHO developed the Global Fistula Map to assess how many providers provided fistula repair services and to provide an illustration of available services for women with fistula [43]. The Map demonstrates that currently, despite increased international attention to the issue, the number of women with fistula is increasing because the number of repairs is less than the number of new cases [22]. It is estimated that up to 80 % of women living with fistula do not receive appropriate care [22, 45]. Given the lack of providers, the International Federation of Gynecology and Obstetrics, published a competency-based fistula-training manual in 2011 [22]. The manual, available on their website at no cost, is meant to promote standardization of surgical training for fistula repair and increased the number of appropriately trained providers able to provide high quality surgical repair [22, 46].

The next question on the topic of training and capacity building is how much money will programs cost, and what results can be expected from the investment. On the issue of what gains can be expected from preventing fistula, a recent paper was published on the surgically avertable burden of obstetric conditions in low and middle-income regions (LMIC) [47]. This manuscript examines five conditions (maternal hemorrhage, obstructed labor, obstetric fistula, abortion, and neonatal encephalopathy) and uses demographic and epidemiological data from the 2010 Global Burden of Disease study to estimate avertable disability adjusted life years (DALYs)—or years of healthy life lost caused by the burden of a disease [47]. The analysis suggests that 37 % of DALYs are avertable by the provision of universal, quality obstetric surgery in LMIC. The study suggests that obstructed labor and obstetric fistula have the highest rates of avertable burden and that sub-Saharan Africa and South Asia carry the largest proportion of this burden [47]. According to these authors, 1,121,346 DALYs (100 % of associated DALYs) and 996,555 DALYs (89 % of associated DALYs) would be averted if quality obstetric surgical services were available to prevent fistula formation [47]. As for cost estimates, it has been suggested that the investment required to scale up provision of comprehensive universal obstetric care through from the prenatal through postnatal period in the 75 LMIC most in need of such care would require 39 billion dollars [48]. For comparison, as of 2012 UNAIDS estimates that 122.5 billion dollars had been invested in the global HIV/AIDS response [49].

### Ethical considerations

Fistula patients deserve high quality care when they present for fistula treatment. As such, a bill of rights for the fistula patient has been developed, as has a code of ethics for the fistula surgeon [50–52]. The bill of rights states that fistula patients should be treated with compassion, dignity, and respect; they should have a right to privacy and to full information and education regarding their condition; they should have the right to direct their own care—including refusal of treatment; and they have the right to receive high quality care, and to be fed, clothed, and sheltered during that process [51]. The code of ethics of the fistula surgeon holds the provider to the standard of providing the highest quality care to the fistula patient and holding her welfare above all else; treating her with dignity, respect, compassion, honesty, and keeping her care confidential; taking responsibility for her total care including appropriate pre-operative and follow-up care; to not experiment on her or provide care in which the surgeon is not trained; to be committed to evidence-based care and to be willing to alter methods based on best-practice guidelines; to not take advantage or allow others to take advantage of the fistula patient (physically, emotionally, economically, sexually); to exercise good stewardship over the financial resources entrusted to them for the care of the fistula patients; to work as part of a fistula care team and obey the laws of the country in which they practice; and to be an advocate on behalf of fistula patients to help remove barriers that hinder access to emergency obstetrical care [50].

### Future research

More research would improve clinical care as well as the full spectrum of supportive care services that accompany fistula treatment, such as psychosocial and economic support, in addition to guiding the training and health system capacity building expected by the global community. That being said, specialists in the field formulated recommendations regarding the clinical topics they feel would most benefit from rigorous controlled trials, which include: the “efficacy/safety of short-term catheterization; efficacy of surgical and nonsurgical therapies for urinary incontinence; technical measures during fistula repair to reduce the incidence of post-surgery incontinence; identification of predictive factors for ‘incurable fistula’; usefulness of urodynamic studies in the management of urinary incontinence; incidence and significance of multi-drug resistant bacteria in the fistula population; primary management of small, new fistulae by catheter drainage; and antibiotic prophylaxis in fistula repair” [53].

## Conclusion

This review has addressed the multifaceted nature of urogenital fistula and that it may be a natural sequela of obstructed labor or iatrogenic. Fistula formation is primarily rooted in poor access to quality labor and delivery care. It deeply affects and disorganizes women’s lives, plaguing the poorest and most marginalized populations, and clinical management of urogenital fistula is in dire need of an evidence-base, despite published guidelines. In conclusion, successful treatment of a fistula patient not only means closure of her fistula, but incorporation into a comprehensive treatment program aimed at achieving continence, mental health, physical rehabilitation, and socioeconomic training and support. Urogenital fistula resulting from childbirth is an historical footnote in HIC, and the provision of high-quality comprehensive emergency obstetrical care in LIC would render it extinct on a global scale. With a manageable price tag of 39 billion dollars, a massive burden of death and disability would be lifted from the human race, resulting in the survival of healthy women and children who bring near limitless potential for their own and the common good.
